# Knowledge of prostate cancer presentation, etiology, and screening practices among women: a mixed-methods systematic review

**DOI:** 10.1186/s13643-021-01695-5

**Published:** 2021-05-06

**Authors:** Ebenezer Wiafe, Kofi Boamah Mensah, Adwoa Bemah Boamah Mensah, Varsha Bangalee, Frasia Oosthuizen

**Affiliations:** 1 University of KwaZulu-Natal, Durban, South Africa; 2 Ho Teaching Hospital, Ho, Ghana; 3 Kwame Nkrumah University of Science and Technology, Kumasi, Ghana

**Keywords:** Awareness, Signs and symptoms, Causes and risk factors, Screening recommendations, Prostate cancer, Women

## Abstract

**Background:**

With the burden of prostate cancer, it has become imperative to exploit cost-effective ways to tackle this menace. Women have demonstrated their ability to recognize early cancer signs, and it is, therefore, relevant to include women in strategies to improve the early detection of prostate cancer. This systematic review seeks to gather evidence from studies that investigated women’s knowledge about (1) the signs and symptoms, (2) causes and risk factors, and (3) the screening modalities of prostate cancer. Findings from the review will better position women in the fight against the late detection of prostate cancer.

**Methods:**

The convergent segregated approach to the conduct of mixed-methods systematic reviews was employed. Five databases, namely, MEDLINE (EBSCOhost), CINAHL (EBSCOhost), PsycINFO (EBSCOhost), Web of Science, and EMBASE (Ovid), were searched from January 1999 to December 2019 for studies conducted with a focus on the knowledge of women on the signs and symptoms, the causes and risk factors, and the screening modalities of prostate cancer.

**Results:**

Of 2201 titles and abstracts screened, 22 full-text papers were retrieved and reviewed, and 7 were included: 3 quantitative, 1 qualitative, and 3 mixed-methods studies. Both quantitative and qualitative findings indicate that women have moderate knowledge of the signs and symptoms and the causes and risk factors of prostate cancer. However, women recorded poor knowledge about prostate cancer screening modalities or tools.

**Conclusions:**

Moderate knowledge of women on the signs and symptoms and the causes and risk factors of prostate cancer was associated with education. These findings provide vital information for the prevention and control of prostate cancer and encourage policy-makers to incorporate health promotion and awareness campaigns in health policies to improve knowledge and awareness of prostate cancer globally.

**Systematic review registration:**

Open Science Framework (OSF) registration DOI: 10.17605/OSF.IO/BR456

**Supplementary Information:**

The online version contains supplementary material available at 10.1186/s13643-021-01695-5.

## Background

Prostate cancer (PCa) is the most common non-skin cancer occurring in men and is accountable for 3.8% of all mortality caused by cancer in men [1, 2]. According to the GLOBOCAN, 2018 database, it is estimated that it is the fifth primary cause of cancer death in men globally. It further reported that the highest mortality rate is found in the Caribbean and Southern African men worldwide [1, 3]. A recent study by Yeboah-Asiamah et al. reported that PCa was the second most common cancer in areas such as Australia, the USA, and New Zealand [4]. Though fewer than 30% of all incidence of PCa are from developing countries, these countries have previously been estimated to have the highest mortality from PCa due to late diagnosis [5, 6]. Although sub-Saharan Africa (SSA) has a low rate of the disease, the incidence is projected to increase if screening is encouraged [7]. Hence, PCa remains a vital public health concern in both developed and developing countries.

The Centers for Disease Control and Prevention (CDC) in North America organized a workshop with the motive to explore strategies to control and prevent the disease based on the increasing incidence and mortality rate of PCa [8]. To address mortality rates related to the disease, participants recommended strategies to improve PCa awareness [8]. Also, as documented by many studies, PCa incidence is a direct reflection of the rate at which high-risk groups screen for the disease [4, 9]. In Europe, early screening was attributed to a 20% reduction in PCa mortality rate [10]. Although there is evidence suggesting a reduction in PCa mortality due to early screening, a United States (US) study did not highlight a reduction in mortality [11]. The prostate-specific antigen (PSA) test and the digital rectal examination (DRE) are useful screening tools, although initial controversies were surrounding the use of these tools [12]. Because of overlap in PSA levels in men with prostatitis, benign prostatic hyperplasia, and PCa, it was assumed that PCa cannot be screened using the PSA test [13]. Catalona et al. demonstrated that PSA could be utilized as a screening tool for PCa, and it has widely been adopted [14]. DRE is the only procedure whereby physicians can examine part of the prostate gland [15]. The findings are only based on the physician impression, hence poor inter-rater reliability and also a limitation to the palpable region of the prostate gland [15]. However, DRE sometimes detects PCa in men with PSA, 4.0 ng/mL [16]. Regardless of the controversial nature of screening and the potential for early screening to reduce mortality, studies support the need to encourage screening [4, 12].

Women have essential characteristics that make them better managers of family health as compared to men. Therefore, it is not surprising that there is evidence positioning women as individuals who make adequate observations about the health of their partners [9, 17]. In promoting the early detection of PCa, women have been documented to observe the slightest symptoms presented by their partners and push them to seek medical attention [9, 18]. In a study conducted by Blanchard et al., it was recommended that efforts must be made to actively involve women in improving the timely detection of PCa through the closure of knowledge gaps [19].

Also, men admit seeking out their wives’ opinions as sources of health information [20]. In the context of the early detection of PCa, women can play various roles such as information seekers, advocates, health advisors, and support persons [21]. Therefore, there is the need to gather current evidence about women’s knowledge of PCa as the findings will be vital in equipping women to contribute towards the early detection of the disease.

In light of the availability of limited evidence addressing the awareness of women on prostate cancer, this review will seek to combine quantitative and qualitative data to increase the validity of findings through data triangulation as recommended by Caruth and supported by Lizarondo et al. [22, 23]. Thus, this review seeks to map out current evidence regarding women’s awareness of PCa under the scopes of (1) signs and symptoms, (2) risk factors and causes, and (3) screening guidelines.

### Review question

Do women have adequate knowledge about prostate cancer?

## Methodology

The Joanna Briggs Institute (JBI) reviewer’s manual for the conduct of mixed-methods critical appraisal and synthesis formed the backbone of the study [23]. With guidance from the JBI manual, a protocol was developed to guide the review process according to the convergent segregated approach [23]. The respective DOIs of the review protocol and review, registered with the Open Science Framework (OSF), are 10.17605/OSF.IO/EYHF2 and 10.17605/OSF.IO/BR456. The review protocol is readily available to the scientific community [24].

### Inclusion criteria

The following were grounds for the inclusion of studies:

- Studies that were conducted among women aged 18 years and above.
- Studies that were conducted among women of all racial backgrounds.
- Studies published from January 1999 to December 2019.
- Studies that were conducted among women of all geographical locations.
- Studies of all research designs.
- Studies that were conducted to investigate the knowledge of women on the signs and symptoms of prostate cancer as highlighted in the review protocol.
- Studies that were conducted to investigate the knowledge of women on the causes and risk factors of prostate cancer as highlighted in the review protocol.
- Studies that were conducted to investigate the knowledge of women on the screening recommendations of prostate cancer as highlighted in the review protocol.
- Studies that were published in the English language.
- Studies with abstract and full text available.

### Exclusion criteria

The following were grounds for the exclusion of studies:

- Studies that were published before January 1999 or after December 2019.
- Studies that were not published in the English language.
- Studies that include women below the age of 18 years.
- Studies in which the age of included women cannot be established.
- Studies that did not indicate the number/percentage of included women.
- Studies that exclusively included men without any women component (18 years and above).
- Studies conducted among women who were previously given education on prostate cancer.
- Studies that exclusively involved lesbian, gay, bisexual, transsexual/transgender, and queer/questioning (LGBTQ) participants.
- Studies that exclusively included healthcare professionals.
- Studies that exclusively involved healthcare and college/university students.
- Studies that do not include the outcome of interest.
- Book chapters.
- Reviews and overviews.
- Abstracts and conference papers.
- Dissertations and thesis.
- Commentaries and letters to editors.
- Studies published without abstracts.

### Information sources and search strategy

An initial explorative search in PubMed founded search terms in preparation for comprehensive electronic search. The selected search terms, applied as MeSH terms, were combined with Boolean operators for a comprehensive electronic search in MEDLINE (EBSCOhost), CINAHL (EBSCOhost), PsycINFO (EBSCOhost), Web of Science, and EMBASE (Ovid) as “(prostate cancer ) AND (awareness OR knowledge) AND (signs OR symptoms) AND (risk factors OR causes) AND (screening) AND (women)”. The search strategy (Additional file 1), so developed, was utilized by the first (EW) and second (KBM) reviewers to independently conduct a literature search as outlined in the review protocol e24].

### Selection of studies

**Fig. 1:**
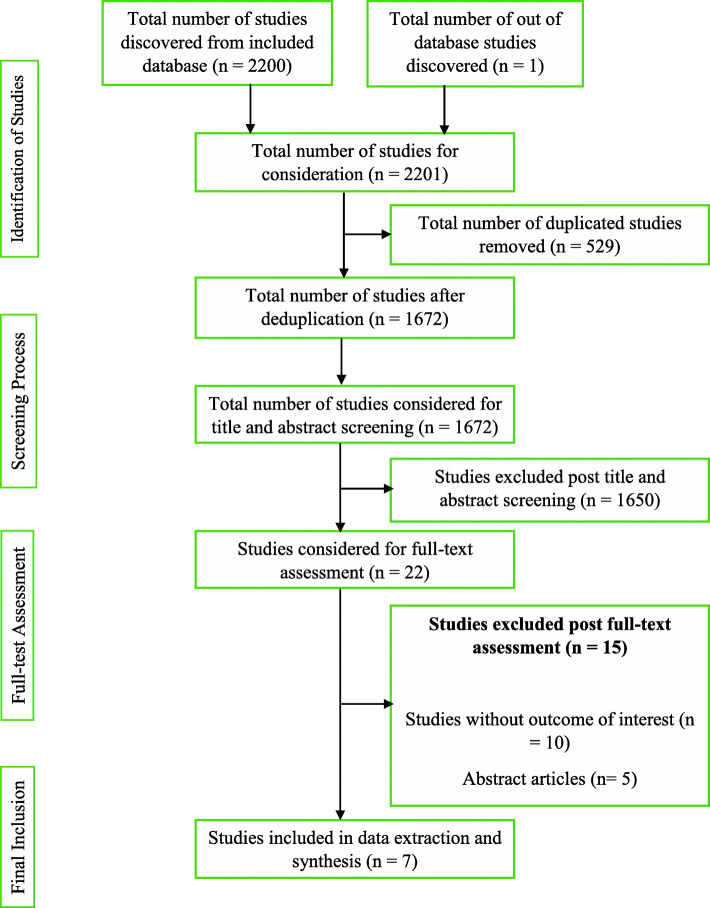
Summary of study selection process

The first and second reviewers, being guided by the developed review protocol, singularly screened and compared the titles and abstracts of the literature search outcomes to a developed standard (the inclusion and exclusion criteria). Studies that successfully passed the initial stage of screening were subjected to the independent full-text reading by EW and KBM before consideration for data extraction. Lastly, hand-searching and snowballing on references of selected articles were done to find eligible studies in the grey area. There were no disagreements between EW and KBM. Hence, the third reviewer (ABBM) assessed the studies before data extraction was conducted by the lead author according to the JBI data extraction tools outlined in the review protocol [24]. The characteristic of studies that successfully went through the data extraction, the key findings that were extracted, and a summary of the study selection process are detailed respectively (Tables 1 and 2 and Fig. 1).

**Table 1:** Characteristics of selected studies

Author and year	Country(s)	Ethnic/cultural background(s)	Study population	Other conditions/domains studied	Research design	Length of study	Sample size of interest population
Blanchard et al., 2005 [19]	USA	Caucasians/Whites, African-Americans/Blacks, Hispanics/Whites, and Hispanics/Blacks	Women	None.	Quantitative study	Missing	324
Brown et al., 2006 [25]	USA	African-Americans and Afro-Caribbeans	Women	Heart health, breast health, prostate health, second-hand smoke, asthma, and sexual health.	Cross-sectional quantitative study	5 days	221
Carrasco-Garrido et al., 2014 [26]	Spain	Spanish	Men and women	Colorectal cancer, breast cancer, and cervical cancer.	Population-based cross-sectional mixed-methods study	2 months	4040 (50.9% of 7938)
Okoro et al., 2018 [27]	USA	Black/African-Americans	Men and women	None.	Cross-sectional mixed methods study	3 months	297
Owens et al., 2015 [18]	USA	African-Americans	Men and women	None.	Mixed methods study	About 2 months	38
Schulman et al., 2003 [28]	France, Germany, Italy, Spain, Sweden, the UK, and the USA	Western Europeans and Americans	Men and women	Breast cancer, lung cancer, bowel cancer, heart disease, stroke, diabetes	Quantitative study (telephone interview)	19 days	700
Webb et al., 2006 [29]	USA	Blacks (non-Hispanics) and Hispanics/Latinos	Men and women	None.	Qualitative study (focus group discussion)	Missing	14

**Table 2:** Summarized study findings

Study title	Findings	Conclusion	Limitations
Knowledge, attitudes and beliefs of women about the importance of prostate cancer screening (Blanchard et al. [19]).	1. The mean score for women’s knowledge about prostate cancer and screening guidelines was determined to be 6.99 ± 3.54 out of 15 points, which were equally scored to reflect knowledge score stratification. 2. Educational level and income were discovered to have increased the mean score for women’s knowledge. 3. Women who disclosed their familiarity with cancer of the prostate and available screening recommendations recorded higher scores in knowledge assessment. 4. Only 54.3% of women knew about the asymptomatic presentation of prostate cancer in the early stages. 5. About 37% of women failed to recognize age as a risk factor for prostate cancer. 6. 83.9% of women were know that men, symptomatic or not, should screen for prostate cancer. 7. 54% of married and 42% of single women recognized the early detection of prostate cancer as the key importance of screening. 8. Married (41%) and single (32%) women agreed that men feared prostate cancer screening results as well as the application of the digital rectal examination for screening.	Women are not knowledgeable about prostate cancer. An educational intervention model, targeting women, could equip women to contribute to the early detection of prostate cancer by encouraging men to screen routinely for the disease.	1. Women might not have documented true responses to questionnaire items since a self-reporting technique was employed in the study. 2. The study was limited to only women fluent in the English language and hence, findings could not be extended to cover all women in New Orleans. 3. The use of the convenience sampling method in the study exposed the study to participants’ selection bias and hence, a negative impact on the generalization of study findings.
Assessment of preventive health knowledge and behaviors of African-American and Afro-Caribbean women in urban settings (Brown et al. [25]).	1. Generally, the knowledge score of women on the symptoms of prostate cancer was appreciable as the mean knowledge score was found to be 20.27 ± 5.51 on a scale of 27. Also, the scores of participants ranged from 6 to 27. 2. Although the knowledge score covered all the domains of medical conditions that were studied, 3 out of the 4 questionnaire items that evaluated women’s knowledge about the symptoms of prostate cancer recorded correct response in 63 to 67% of women. 3. Women who knew about the existence of prostate cancer in their families had higher knowledge scores. 4. 24% of women responded that prostate cancer is asymptomatic; whilst 65%, 67%, and 63% respectively noted the difficulty in passing urine, dysuria, and the need to frequently pass urine as symptoms. 5. Women found it difficult in identifying tools applicable to prostate cancer screening. 6. 46%, 61%, and 38% of women respectively selected prostate-specific antigen (PSA), digital rectal examination (DRE), and x-ray as prostate cancer screening tools.	Women are more knowledgeable about the symptoms of prostate cancer but know very little about prostate cancer screening tools. An intervention is needed to upgrade the knowledge of women on the symptoms and screening tools applicable to prostate cancer.	1. The study suffered various forms of selection bias as the participants were conveniently selected from salons that were interested in the health promotion initiatives of the Arthur Ashe Institute for Urban Health (AAIUH). 2. The study was restricted to women who used the services of the selected salons and hence, the study findings could not be a true reflection of all New York women. 3. There was an observation of a high number of correctly answered questions.
Awareness and uptake of colorectal, breast, cervical, and prostate cancer screening tests in Spain (Carrasco-Garrido et al. [26]).	1. 51.56% of Spanish women knew PSA as a prostate cancer screening tool. 2. Education and social status significantly increased women’s awareness of PSA as a prostate cancer screening tool.	The use of prostate-specific antigen (PSA) for prostate cancer screening is poorly known to women. Women should be comprehensively educated on screening tools.	1. The validity and reliability of the survey instrument were not done in the study population. 2. Respondents might have given socially acceptable responses when their awareness about PSA was tested. 3. Women who knew about PSA as a prostate cancer screening tool might have been high in the study. 4. Knowledge scores were not adequately stratified or described.
Leveraging the family influence of women in prostate cancer efforts targeting African American Men (Okoro et al .[27]).	1. Although knowledge scores were not stratified, on a 25 knowledge-score scale, women’s mean score was 11.4 ± 5.1. 2. No idea accounted for 29.1% of women’s responses to prostate cancer knowledge. 3. The focused group discussion involving women revealed an overall poor prostate cancer knowledge. 4. The PSA as a prostate cancer confirmatory tool and the recommended age for universal prostate cancer screening received the worst correct response rates. 5. Only 17.5% of women knew elevated PSA levels did not exclusively indicate the existence of prostate cancer. 6. As low as 13.5% of women knew universal prostate cancer screening is not exclusively a recommendation for only men above 50 years. 7. The educational status of women greatly increased knowledge scores. 8. 62.3%, 57.2%, and 38.7% of women respectively identified the presence of a first-degree relative, being a man of African descent, and excessive truncal obesity as risk factors of prostate cancer. 9. Women (54.5%) knew the asymptomatic nature of prostate cancer. 10. 47.5% of women recognized DRE as a tool for the early detection of prostate cancer. 11. Women (40.7%) indicated the need for risk assessment before the initiation of prostate cancer screening, whilst 54.2% agreed with the recommendation that men who are 40–45 years and are at risk for the development of the disease should seek adequate health information from registered healthcare providers.	The knowledge and awareness of women about prostate cancer are not appreciable. An educational intervention model can increase prostate cancer awareness and knowledge among women.	1. The study included only African-American women and hence, findings cannot be extended to cover all women in America. 2. The study suffered selection bias as participants were conveniently sampled. 3. The survey instrument did not undergo validation and reliability assessment in the study population. 4. The study engaged relatively young participants and hence findings could not be an exact representation of all age groups. 5. The study participants, being young, might have accounted for the observed low knowledge scores. 6. Knowledge scores were not adequately stratified or described.
Prostate cancer knowledge and decision making among African-American men and women in the southeastern United States (Owens et al. [18]).	1. Women had limited knowledge about prostate cancer. 2. The only signs and symptoms of prostate cancer women were conversant with included urinary frequency, difficulty in urinating, an enlarged prostate gland, and erectile dysfunction. 3. Women acknowledged knowing very little about prostate cancer and called for education. 4. Most women did not know the location of the prostate gland in addition to the available screening tools. Nevertheless, the PSA was mentioned. 5. Some women perceived colonoscopy as a prostate cancer screening tool. 6. Risk factors that attracted much attention from women included; poor diet (high red meat and fatty food consumption) and inadequate physical activity. 7. Other risk factors that did not attract much attention included increased age (where age greater or equal to 45 years was tagged the highest risk), stressful lifestyle, family history of the disease, being of African decency, poor screening habit, cigarette smoking, and poor access to quality healthcare. 8. Women erroneously perceived a man’s sexuality and regularity of sexual intercourse as risk factors.	The knowledge of women on prostate cancer was minimal. With education on prostate cancer, women’s knowledge was improved. There is a need for a community-based public health intervention geared towards educating women on prostate cancer.	1. The relatively small sample size of the interest population hindered the results’ generalizability. 2. The study was limited to African-Americans and hence, findings could not be generalized to cover other races/ethnic diversities in the study site. 3. The participants were conveniently sampled and hence, the poor generalizability of results.
Awareness of prostate cancer among the general public: findings of an independent international survey (Schulman et al. [28]).	1. 100 women each from 7 countries were involved in the study. 2. 28% of female respondents spontaneously included prostate cancer in their list of available cancers whilst 69%, who did not initially list prostate cancer, agreed to the existence of the disease when asked a closed-ended question. 3. Women in the UK (40%), USA (20%), France (23%), Germany (24%), Italy (21%), Spain (26%), and Sweden (39%) were spontaneously aware of prostate cancer. When prompted, additional respective 58%, 76%, 70%, 75%, 76%, 69% and 61% of women recognized the existence of prostate cancer. 4. Women in Spain (36%), the USA (35%), Italy (23%), Sweden (22%), the UK (17%), France (17%), and Germany (9%) recognized PSA as a prostate cancer screening tool. 5. 20% of women in the USA, 14% in France, 8% in Spain, 6% in the UK, 6% in Germany, 5% in Italy, and 2% in Sweden recognized DRE as a prostate cancer screening tool. 6. Mistakenly, 37% of women in Spain, 22% in Italy, 17% in France, 13% in the UK, 10% in Germany, 11% in Sweden, and 5% in the USA recognized the use of urine as a prostate cancer screening sample. 7. The inability of women to recognize at least a prostate cancer screening tool followed the trend: Germany (71%), Sweden (60%), the UK (56%), the USA (53%), France (52%), Italy (44%), and Spain (41%).	The recognition of the basic prostate cancer screening tools by women was very low. The general awareness of prostate cancer was lacking in women. To promote the early detection of prostate cancer in an attempt to reduce mortality and educational intervention, targeting women is needed.	1. The study failed to indicate the percentage of women who were able to identify the signs and symptoms, and risk factors of prostate cancer. 2. The number of participants from the various countries was relatively small to promote the generalizability of the results. 3. Respondents might have given socially approved responses since data collection was through a telephone interview. 4. The validity and reliability of the questionnaire were not determined in the study population. 5. Knowledge scores were not adequately stratified or described.
An evaluation of the knowledge, attitudes, and beliefs of African-American men and their female significant others regarding prostate cancer screening (Webb et al. [29]).	1. Women disclosed that prostate cancer may occur in men who are or greater than 65 years old. However, women were not sure if a diet has caused a reduction in the age at which men develop prostate cancer. 2. During the FDG, some women agreed that prostate cancer screening starts when men celebrate their 40th birthday. 3. The use of blood as a screening sample for prostate cancer detection was mentioned by women. However, women reported the need for a physical body examination in addition to blood analysis.	The knowledge women possess about prostate cancer screening has appreciable gaps. Educating women on prostate cancer screening is of equal importance as compared to male prostate cancer education.	1. Results have low generalizability due to the utilization of the convenience sampling strategy. 2. Validity and reliability studies of the FGD questions were not done in the study population. 3. The target number of study subjects needed for the FDG was not met. Hence, the study sample was inadequate.

### Quality assessment

As described in the review protocol [24], the methodological quality assessment tool (Additional file 2) was adopted and modified for this review due to the similarities this review shares with the study conducted by Mensah et al. [30]. The tool appraised the studies’ quality based on the study sample representativeness, response rate, reliability, and validity of the data collection tool. The tool was modified to suit the results from the included studies. A score was calculated, and the quality of the studies was classified as weak (0 to 33.9%), moderate (34 to 66.9%), or strong (67 to 100%). Eligible records were subjected to independent quality assessment by EW and KBM. Methodological quality outcomes were not grounds for exclusion.

### Synthesis and integration of findings

The review findings were subjected to the convergent segregated approach to synthesis and integration according to the developed review protocol [24]. A narrative synthesis was separately performed for qualitative and quantitative findings. The heterogeneous nature of the review findings did not support the conduct of a meta-analysis. The results were finally integrated.

## Results

Conducting the review, according to the developed protocol, yielded 2200 study results. A detailed citation screening led to an additional study, which increased the total number of studies to 2201. Regarding the summary of the study selection process (Fig. 1), 1672 studies were obtained after 529 duplicates were removed from the pool of data. Post-titles and abstract review excluded 1650 studies leaving 22 studies. The 22 studies were further reduced to 7 after a full-text reading resulted in the exclusion of 15 studies.

### Characteristics of included studies

The data extracted from the seven (7) studies are detailed (Table 1). The publication years ranged from 2003 to 2018 with 5 studies having been conducted in the USA. One of the studies was a multicenter study that involved multinationals [28]. The study with the highest female participants (4040 women) was conducted in Spain [26]. Webb et al. recruited the lowest sample size, 14 women [29]. A total of 5634 women were involved in the 7 studies. Two studies were solely conducted in women, three included other diseases, and two did not disclose study duration.

### Quality of included studies

According to the scoring scheme of the quality assessment tool (Additional file 2), two studies [26, 29] were evaluated as moderate quality whilst five studies were evaluated as strong quality. None of the studies was excluded based on methodological quality assessment outcomes. There was no disagreement between EW and KBM.

### Review findings

Study findings, presented in Table 2, were heterogeneous. Quantitative studies indicate that women knew about the existence of PCa. In exploring qualitative evidence, women exhibited knowledge of PCa. Therefore, both arms of the review are supportive of each other.

Women had moderate knowledge about the signs and symptoms of PCa drawing from quantitative findings. Women knew about the asymptomatic nature of the early stages of PCa. They also moderately knew urinary symptoms such as urinary frequency, difficulty in urinating, and dysuria. Qualitative studies indicate that women were aware prostate cancer patients, usually in advanced stages, could present with signs and symptoms such as urinary frequency, difficulty in urinating, glandular enlargement of the prostate, and erectile dysfunction. Hence, quantitative and qualitative findings revealed that women moderately knew the urinary symptoms of PCa.

Quantitative studies indicate an average score of women on knowledge of risk factors of PCa. Risk factors women knew were increasing age, presence of a first-degree relative, being genetically linked to Africa, and excessive truncal obesity. Qualitative evidence recognized all risk factors documented by the quantitative findings except truncal obesity. Also, identified risk factors included poor diet, inadequate exercise, stressful lifestyle, poor screening habits, cigarette smoking, and poor access to quality healthcare. Women wrongly reported sexual orientation and frequent sexual activity as risk factors. Therefore, qualitative findings confirm the quantitative claim that women have shared knowledge about the risk factors of PCa.

Quantitative studies indicate that women had poor knowledge about PCa screening guidelines, appropriate screening samples, and tools. Although it was reported that women knew about PSA and DRE, the proportions of women who had correct responses to screening knowledge items were not appreciable. Women poorly recognized urine as a screening sample, PSA as an exclusive diagnostic tool (where only 17.5% answered correctly), and failed to identify more than one screening tool (between 41 and 71% of women failed). Qualitative studies respectively reported PSA and blood as a screening tool and sample. Colonoscopy was wrongly reported as a PCa screening tool. Conclusively, both arms of the review reported women knew about PSA and had poor knowledge about PCa screening.

## Discussion

The heterogeneity of the study findings warranted the synthesis as a narrative [23, 31]. The convergent segregated approach was employed according to the recommendation of the JBI reviewer’s manual [23].

Generally, from the quantitative evidence, women knew about prostate cancer [19, 25, 27, 28]. The knowledge of women was found to have increased with educational and financial status [19], and disease familiarity [19, 25]. The awareness of women about the existence of PCa increased when the disease was mentioned compared to an initial request for women to list cancers [28]. Qualitative evidence showed that women were aware of PCa [18, 27]. They appreciated and specifically requested for PCa education partly because they could not tell the location of the prostate gland [18]. Thus, quantitative and qualitative evidence indicates that women know about PCa. Women’s awareness could be due to their role in family health management and the possible health-seeking behavior of educated and financially strong women. As persons are faced with the experiences of a health condition, they will seek to make sense of this illness by acquiring knowledge [32], experiences, and beliefs; hence, this theory might explain the improved awareness of women who were familiar with the disease.

Most of the quantitative studies indicate that women are aware of the asymptomatic nature of early-stage PCa [19, 25, 27]. Symptoms that women had a fair knowledge about included urinary frequency, difficulty in urinating, and dysuria [25]. Findings from one of the qualitative studies indicate that women fairly recognized urinary frequency, difficulty in urinating, glandular enlargement of the prostate, and erectile dysfunction as signs and symptoms of PCa [18]. Being familiar with the disease may explain the awareness of women of the urinary symptoms associated with PCa.

According to Okoro and colleagues’ quantitative study, although knowledge of PCa was not adequate, women knew associated risk factors such as being a first-degree relative, being a man of African descent, and excessive truncal obesity [27]. Blanchard et al. also documented women’s recognition of increasing age as a PCa risk factor [19]. One of the qualitative studies indicates women knew increasing age could increase a man’s chance for PCa development [18, 29]. Other causes and risk factors women identified included poor diet, inadequate exercise, stressful lifestyle, family history of the disease, being of African descent, poor screening habits, cigarette smoking, and poor access to quality healthcare [18]. Erroneously, one study reported that women perceived sexual orientation and frequent sexual activity as risk factors [18]. Both quantitative and qualitative findings documented women knew increasing age, family history, and African descent as PCa risk factors.

Quantitatively, women’s responses to queries about PCa screening were poor [25, 28]. Some women were unable to recognize at least a PCa screening tool whilst others mistakenly recognized urine as a suitable sample for PCa screening [28]. According to Okoro et al., the majority of women exclusively tagged PSA elevation as a basis for PCa diagnosis [27]. This, therefore, calls for extensive education because benign prostatic hyperplasia, prostatitis, and PCa usually present with elevated PSA [13]. Evidence from qualitative findings indicated women knew physical examination must augment blood analysis [29]. Also, women mentioned PSA and colonoscopy as screening tools [18]. The results from included qualitative studies confirmed that women had poor knowledge about PCa screening. The mention of colonoscopy as a screening tool further supports a lack of adequate knowledge about PCa screening.

This critical appraisal and synthesis revealed over the 20 years of study search, only four studies out of the seven included studies investigated all the outcomes of interest. Two studies did not investigate women’s awareness of the signs and symptoms [26, 29] and the causes and risk factors [25, 26] of PCa. Therefore, although quantitative and qualitative findings were supportive of each other, studies investigating the causes and risk factors, as well as the signs and symptoms of PCa, were lacking.

## Recommendations for practice

From the review findings, it is recommended that PCa control programs should also focus on educating women. Clinicians and public health practitioners should include women in prostate cancer health promotion. Women should be encouraged to attend PCa clinics with their male significant others suffering from the disease, and the effect of this strategy in reducing PCa mortality rate must be investigated.

## Recommendations for research

Further studies are recommended to investigate the knowledge of women living in low- and middle-income countries (LMIC) about PCa. Such studies should focus extensively on the knowledge of women on PCa screening. Also, it is recommended for research to develop and pilot a PCa educational intervention model, applicable to women to reduce the burden of the disease. This tool should be culturally specific for easy acceptance and recognition. Also, current evidence on the willingness of women to offer social support to men with PCa should be investigated.

## Study limitations

The various restrictions that were imposed on the literature search included a search range from January 1999 to December 2019, a search into only 5 databases, and the outright exclusion of non-English publications. These constitute selection bias. Therefore, some important studies could have been left out of the review.

Although five (5) out of the seven (7) included studies explicitly indicated recruiting participants of African backgrounds, none of the studies were conducted in Africa. Hence, the global generalizability of the review findings, to most importantly cover low and middle-income countries, cannot be documented.

The exclusion of studies conducted in women who received education on prostate cancer, healthcare professionals, healthcare students, and college/university students, and further exclusion of studies that involved (LGBTQ) participants further constitute selection bias.

It is imperative to note that the various limitations, in connection to the included studies, documented in Table 2 have an effect on this review and, as such, could be considered as potential limitations.

## Supplementary Information

**Additional file 1:.** Proposed search strategy using Medline via EBSCOhost

**Additional file 2:.** Assessment of methodological quality of included studies

## Data Availability

Data and other pieces of information are available at 10.17605/OSF.IO/BR456

## Abbreviations

CDC: Centers for disease control and prevention
DRE: Digital rectal examination
GLOBOCAN: Global cancer incidence, mortality and prevalence
JBI: Joanna briggs institute
LGBTQ: Lesbian, gay, bisexual, transsexual/transgender, and queer/questioning
OSF: Open science framework
LMIC: Low- and middle-income countries
PCa: Prostate cancer
PSA: Prostate-specific antigen
SSA: Sub-Saharan Africa
US: United States
