# Supplementary material for: Knowledge of prostate cancer presentation, etiology, and screening practices among women: a mixed-methods systematic review
Source: Syst Rev. 2021 May 6;10:138. doi: 10.1186/s13643-021-01695-5 (PMC8103622; doi:10.1186/s13643-021-01695-5)
Supplement: Supplementary file 2 — Additional file 2:. Assessment of methodological quality of included studies [file 13643_2021_1695_MOESM2_ESM.docx]

**APPENDIX 2**

**ASSESSMENT OF METHODOLOGICAL QUALITY OF INCLUDED STUDIES**

| SN OF SELECTED STUDY | GENERAL QUALITY ASSESSMENT CRITERIA (GQAC) | | | | | SPECIFIC QAC | | | PERCENTAGE SCORE |
| --- | --- | --- | --- | --- | --- | --- | --- | --- | --- |
|  | A | B | C | D | E | F | G | H |  |
| 1.(Blanchard et al., 2005) | 1 | 1 | 1 | 1 | 1 | 1 | 1 | 1 | 100 |
| 2.(Brown et al., 2006) | 1 | 1 | 1 | 1 | 1 | 1 | 0 | 1 | 87.5 |
| 3.(Carrasco-Garrido et al., 2014) | 1 | 1 | 0 | 0 | 1 | 0 | 0 | 1 | 50 |
| 4.(Okoro, Rutherford, & Witherspoon, 2018) | 1 | 1 | 0 | 0 | 1 | 1 | 1 | 1 | 75 |
| 5.(Owens, Jackson, Thomas, Friedman, & Hébert, 2015) | 1 | 0 | 1 | 1 | 1 | 1 | 1 | 1 | 87.5 |
| 6.(Schulman, Kirby, & Fitzpatrick, 2003) | 1 | 1 | 0 | 0 | 1 | 1 | 1 | 1 | 75 |
| 7.(Webb, Kronheim, Williams, & Hartman, 2006) | 1 | 0 | 0 | 0 | 1 | 0 | 1 | 1 | 50 |

**KEY**

SN = Serial Number

GQAC = General Quality Assessment Criteria

QAC = Quality Assessment Criteria

A = Was the study population adequately represented by the selected study sample size?; B = Did the study document a response rate?; C = Did the data extraction instrument undergo a reliability assessment?, D = Did the data extraction instrument undergo a validity assessment?; E = Does this study contribute to a primary source of data?; F = Was the knowledge of women on the signs and symptoms of prostate cancer assessed?; G = Was the knowledge of women on the causes and risk factors of prostate cancer determined? H = Was the knowledge of women on the availability of screening guides for prostate cancer detection ascertained?

**SCORING SCHEME**

Yes (Y) = 1; No (N) or Unclear (U) or Not Reported (NR) = 0

PERCENTAGE SCORE = 100((Selected Study Total Scores) ÷ (Sum of Assessment Criteria Scores))

INTERPRETATION OF SCORES: Weak = 0 – 33.9%, Moderate = 34% – 66.9%, Strong = 67% – 100%
